# Global and Regional Burden of Bacterial Antimicrobial Resistance in Urinary Tract Infections in 2019

**DOI:** 10.3390/jcm11102817

**Published:** 2022-05-17

**Authors:** Xuhui Li, Hua Fan, Hao Zi, Hankun Hu, Binghui Li, Jiao Huang, Pengcheng Luo, Xiantao Zeng

**Affiliations:** 1 Center for Evidence-Based and Translational Medicine, Zhongnan Hospital of Wuhan University, Wuhan 430071, China; xuhuili2011@hotmail.com (X.L.); zihao0828@126.com (H.Z.); dclibinghui@126.com (B.L.); huangjiao1019@163.com (J.H.); 2 Office of Research & Innovation, The First Affiliated Hospital, College of Clinical Medicine of Henan, University of Science and Technology, Luoyang 471000, China; fanhua19851229@126.com; 3 Department of Urology, Institute of Urology, Zhongnan Hospital of Wuhan University, Wuhan 430071, China; 4 Department of Pharmacy, Zhongnan Hospital of Wuhan University, Wuhan 430071, China; huhankun@whu.edu.cn; 5 Department of Urology, Tongren Hospital and Wuhan Third Hospital of Wuhan University, Wuhan 430074, China

**Keywords:** antimicrobial resistance, urinary tract infection, antibiotic, pathogen

## Abstract

Background: There are still no detailed data about the burden of bacterial antimicrobial resistance (AMR) in urinary tract infections (UTI). Concrete knowledge of global and regional bacterial AMR data is crucial for developing informed programs and policies to control bacterial AMR and for prudent use of antibiotics to optimize antibiotic therapy in patients with UTI. This study aimed to provide comprehensive global and regional estimates for the AMR burden of UTI in 2019. Methods: Data were obtained from the Global Burden of Diseases, Injuries, and Risk Factors Study (GBD), including death, disability-adjusted life-years (DALYs), year lived with disability (YLD), and years of life lost (YLL) for bacterial AMR in UTI for 7 GBD super-regions, 21 regions, 14 pathogens, 13 antibiotic classes, and 66 pathogen-antibiotic combinations in 2019. The estimates were based on two counterfactual scenarios: drug-susceptible infection and no infection. Results: Globally, there were 64.89 thousand deaths (95% uncertainty interval [UI]: 45.86–93.35) attributed to and 0.26 million deaths (95% UI: 0.18–0.36) associated with bacterial AMR in UTI in 2019. Among regions, the all-age death rates were higher in southern Latin America, tropical Latin America, and Europe and lower in sub-Saharan Africa. *Escherichia coli* and *Klebsiella pneumoniae* accounted for more than 50% of deaths attributable to and associated with AMR, and resistance was high among multiple types of antibiotic class, including fluoroquinolones, carbapenems, and third-generation cephalosporins. There were 2 pathogen-drug combinations that caused more than 6000 resistance-attributable deaths: third-generation cephalosporin-resistant *Escherichia coli* and fluoroquinolone-resistant *Escherichia coli*. Conclusions: AMR in UTI is an unignorable health problem, both for the management of urology disease and for global antibiotic resistance. Special tailored strategies, including enhanced surveillance and rational use of antibiotics, should be developed for different regions according to the region-specific pathogen-antibiotic situations and resources.

## 1. Introduction

Bacterial antimicrobial resistance (AMR) is a major threat to human health and well-being worldwide that requires global and urgent action to address [1,2]. It causes an enormous and additional burden for patients reflected in extended hospital stays, increased payments, and even needless deaths [3]. The Global Burden of Diseases, Injuries, and Risk Factors Study (GBD) estimated that the number of deaths attributed to bacterial AMR in 2019 reached 1.27 million, which is much larger than the global HIV deaths and malaria deaths and ranks only behind COVID-19 and tuberculosis in terms of infection-related deaths [4,5]. In clinical practice, high rates of resistance to antibiotics, which are often used to treat bacterial infections including urinary tract infections, sepsis, and sexually transmitted infections, have been observed globally [2].

Urinary tract infections (UTI) are among the most common bacterial infections leading to clinic visits and can cause serious sequelae, such as renal damage and urosepsis [6,7,8]. In 2019, the global incident cases of UTI were 0.4 billion, an increase of 60.40% from 1990 [9]. Consistent with the rapid increase in the UTI burden, the ominously rising burden of bacterial AMR in UTI has also been observed. Comprehensively estimating the burden of resistance is of paramount importance in tackling AMR, and there exists extensive literature estimating the burden. For example, a study analyzing data from the Global Prevalence of Infections in Urology (GPIU) study from 2003 to 2010 reported that all antibiotics except imipenem had resistance rates of more than 10% to the total bacterial spectrum [10]. Another study using GPIU data from Asia found that from 2004 to 2013, cephalosporins (34.4%) and fluoroquinolones (24.1%) were the most frequently used antibiotics for UTI, and fluoroquinolone (ciprofloxacin 54.9%, levofloxacin 39.0%) and cephalosporin (42%) resistance were relatively high [11]. A retrospective cohort study from 2013 to 2018 in the US revealed that triple resistance to carbapenems occurred in 1 in 8 patients hospitalized with complicated UTI [12]. These studies provide valuable information about the burden of bacterial AMR in UTI in different countries and regions. However, the results varied substantially across studies and were insufficient for direct comparison among different areas or pathogens. There is still a lack of comprehensive information about the global, location-specific, and pathogen-specific burden of bacterial AMR in UTI.

Concrete knowledge of regional antibiotic resistance data is crucial for developing informed and location-specific programs and policies to control bacterial AMR and for prudent use of antibiotics to optimize antibiotic therapy in patients with UTI. In this study, using data obtained from the updated GBD study, we comprehensively present the global, regional, pathogen-specific, and pathogen-antibiotic combination-specific burden of bacterial AMR in UTI.

## 2. Materials and Methods

### 2.1. UTI Definition

In GBD 2019, UTI was defined as kidney infection that can lead to systemic symptoms such as fever and weakness and can cause discomfort and difficulty with daily activities, including nephritis, cystitis, and urethritis. The ICD-10 codes for UTI are N10, N10.0, N10.9, N11, N11.0, N11.1, N11.8, N11.9, N12, N12.0, N12.9, N13.6, N15, N15.1, N15.8, N15.9, N16, N16.0–N16.5, N16.8, N30, N30.0–N30.3, N30.8–N30.9, N34, N34.0–N34–3, and N39.0 [9,13].

### 2.2. Data Source

Data analyzed in this study were obtained from the GBD study [4], which comprehensively analyzed the death and disability-adjusted life-years (DALYs) attributable to and associated with bacterial AMR for 12 major infectious syndromes (such as bloodstream infections, peritoneal and intra-abdominal infections, urinary tract infections, and pyelonephritis), 23 pathogens (such as *Escherichia coli*, *staphylococcus aureus*, and *Klebsiella pneumoniae*), 18 antibiotic classes (such as carbapenems, aminoglycosides, and fluoroquinolones), and 88 pathogen-drug combinations in 204 countries and territories in 2019.

Data about AMR were obtained from a variety of sources, including multiple causes of death and vital registration records, hospital discharges, microbiology lab results from hospitals, pharmaceutical sales, and national and multi-national surveillance networks. Through data processing and mapping, and intermediate cause and infectious syndrome hierarchy mapping, a total of 471 million personal records or isolates covering 7585 study-location-years collected were used to construct statistical models.

### 2.3. Statistical Analysis

The models were constructed through ten steps to estimate the impact of AMR in all regions, including places without data. The details of the methodology of the GBD study can be found in previous studies [4,13]. As it is difficult to determine whether the drug resistance was a direct or indirect cause of death or loss of DALYs, estimates were made based on two counterfactual scenarios: drug-susceptible infection and no infection. For the drug-susceptible infection scenario, all drug-resistant infections were replaced by drug-sensitive infections, and deaths and DALYs directly attributable to the drug resistance were estimated. For the no infection scenario, all drug-resistant infections were replaced by no infection, and deaths and DALYs associated with resistant infection were estimated. Through this approach, the study calculated the burdens directly and indirectly caused by AMR and addressed the problem of to what extent would drug-resistant infections be replaced by susceptible infections or by no infection.

In this study, we present detailed global, super-regional, and regional estimates for bacterial AMR in UTI for both genders and all ages in 2019 based on the 14 pathogens, 13 antibiotic classes, and 66 pathogen-antibiotic combinations (the list of GBD location hierarchy was shown in Table S1).

## 3. Results

### 3.1. Global and Regional Burden of Bacterial AMR in UTI

Globally, 64.89 thousand deaths (95% uncertainty interval [UI]: 45.86–93.35) with a death rate of 0.84 per 100,000 (95% UI: 0.59–1.22) were directly attributed to and 0.26 million deaths (95% UI: 0.18–0.36) with a death rate of 3.31 per 100,000 (95% UI: 2.36–4.70) were associated with bacterial AMR in 2019. The estimated counts and rates of DALYs were 1.44 million (95% UI: 1.02–2.10) and 18.60 per 100,000 (95% UI: 13.17–27.16), respectively, that were attributed to resistance and 5.61 million (95% UI: 4.08–7.99) and 72.48 per 100,000 (95% UI: 52.75–103.28), respectively, that were associated with resistance. In the 7 GBD super-regions, South Asia had the highest death counts (20.07 thousand [95% UI: 14.56–27.12] attributed to resistance and 72.12 million [95% UI: 53.06–96.02] associated with resistance). However, Central Europe, eastern Europe, and central Asia had the highest death rates (1.45 per 100,000 [95% UI: 0.96–2.23] attributed to resistance and 5.72 per 100,000 [95% UI: 3.84–8.59] associated with resistance). Similarly, South Asia had the highest DALY counts, and Central Europe, eastern Europe, and central Asia had the highest DALY rates (Table 1).

**Figure 1:**
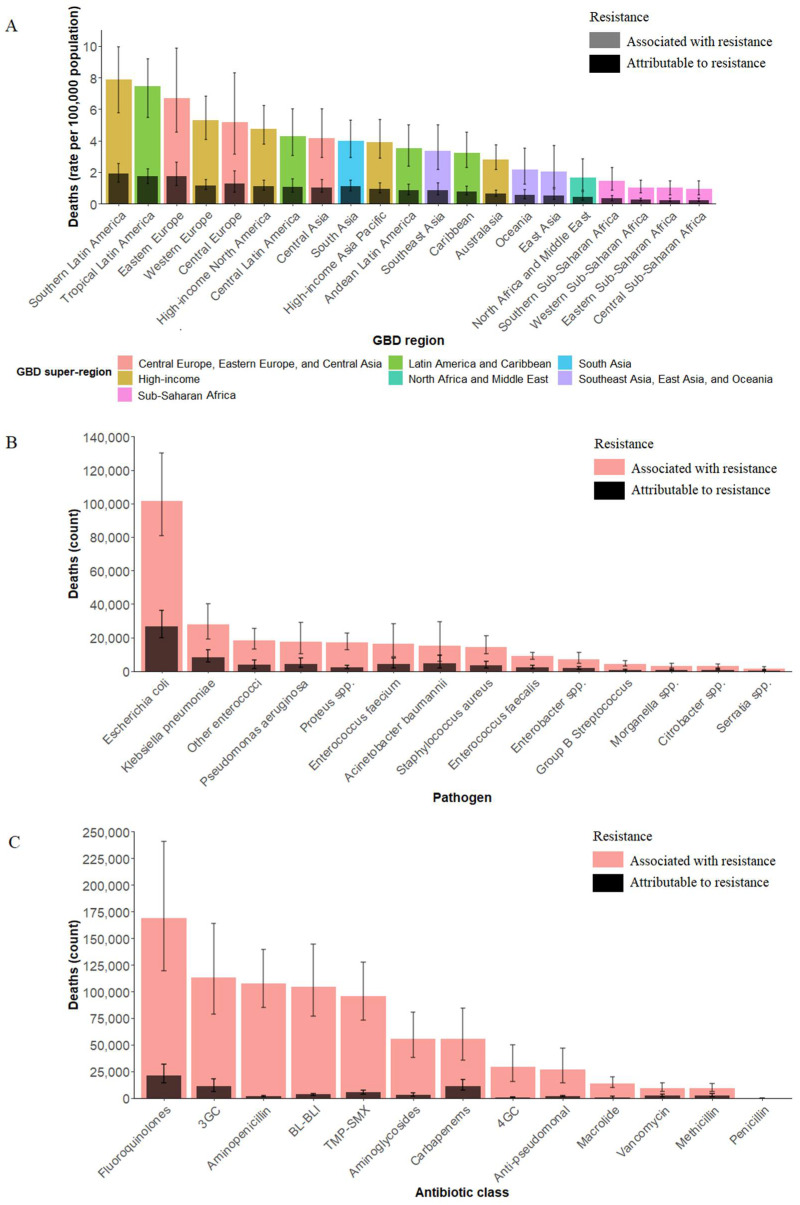
All-age rate of deaths among 21 GBD regions. (**A**) All-age count of deaths among 14 pathogens and (**B**) all-age count of deaths among 13 antibiotic classes (**C**) attributable to and associated with bacterial AMR, 2019. AMR = antimicrobial resistance. GBD = Global Burden of Diseases, Injuries, and Risk Factors Study. 3GC = third-generation cephalosporins. 4GC = fourth-generation cephalosporins. Anti-pseudomonal = anti-pseudomonal penicillin or beta-lactamase inhibitors. BL-BLI = β-lactam or β-lactamase inhibitors. Resistance to 1+ = resistance to one or more drugs. TMP-SMX = trimethoprim/sulfamethoxazole. AMR = antimicrobial resistance. Error bars indicate 95% uncertainty intervals.

Among the 21 GBD regions, southern Latin America (1.91 per 100,000 [95% UI: 1.38–2.53] attributed to resistance and 7.86 per 100,000 [95% UI: 5.76–9.96] associated with resistance), and tropical Latin America (1.74 per 100,000 [95% UI: 1.27–2.22] attributed to resistance and 7.47 per 100,000 [95% UI: 5.46–9.18] associated with resistance) were the two regions with the highest death rates, for the rates both attributable to resistance and associated with resistance. Then, the ranking is followed by three European regions (eastern Europe, western Europe, Central Europe). The regions of sub-Saharan Africa, including southern sub-Saharan Africa, western sub-Saharan Africa, eastern sub-Saharan Africa, and central sub-Saharan Africa, had the lowest death rates (Figure 1A). In terms of DALY rates, the four sub-Saharan Africa regions also had the lowest rates. However, the three regions with the highest DALY rates changed to eastern Europe, tropical Latin America, and Central Asia (Figure S1).

### 3.2. Pathogen-Specific Burden of Bacterial AMR in UTI

In 2019, *Escherichia coli* dominated the global AMR-related deaths in UTI, accounting for about 40%, of which 26.52 thousand [95% UI: 19.79–36.30]) deaths were attributable to AMR and 101.52 thousand [95% UI: 80.65–130.29] were related to AMR. *Klebsiella pneumoniae* was the second most common pathogen responsible for the AMR-related deaths in UTI, with more than 10% of deaths attributed to AMR (8.33 thousand [95% UI: 5.48–12.47]) and associated with AMR (27.68 thousand [95% UI: 19.01–40.31]) (Figure 1B). Similar to the indicator of death number, *Escherichia coli* and *Klebsiella pneumoniae* were the two main pathogens, causing more than half of DALYs attributed to and associated with AMR (Figure S2).

**Figure 2:**
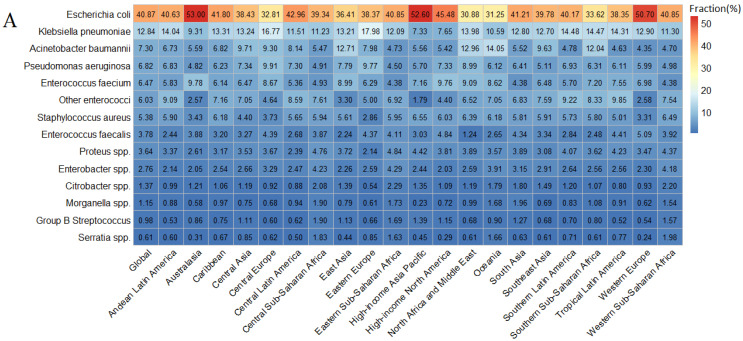

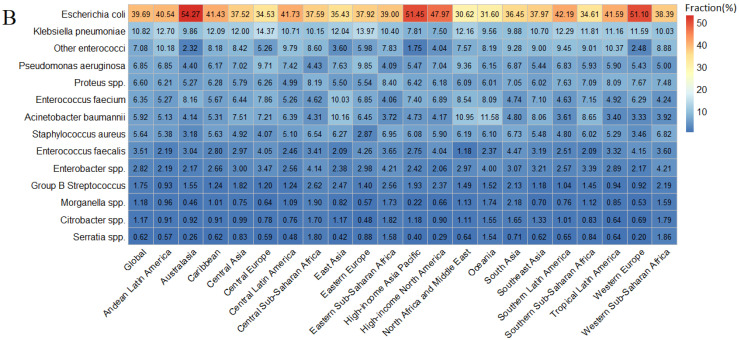
Fraction of deaths attributable to (**A**) and associated with (**B**) bacterial AMR among 21 GBD regions and 14 pathogens, 2019. AMR = antimicrobial resistance. GBD = Global Burden of Diseases, Injuries, and Risk Factors Study.

Similar to the AMR burden proportion of pathogens on the global level, *Escherichia coli* and *Klebsiella pneumoniae* ranked first and second among almost all 21 GBD regions in terms of the fractions of AMR deaths caused by 14 pathogens (Figure 2). However, in Australasia and high-income North America, the bacteria with the second-highest proportion of death attributable to AMR was *Enterococcus faecalis*, while in Oceania, the bacteria with the second-highest proportion of death, either attributable to resistance or associated with resistance, was *Acinetobacter baumannii*. The shares of AMR burden caused by the same pathogen differed substantially across GBD super-regions. For the share of AMR deaths caused by *Escherichia coli*, it ranged from the highest in Australasia (53.00% of deaths attributable to resistance and 54.27% of deaths associated with resistance) to the lowest in North Africa and Middle East (30.88% of deaths attributable to resistance and 30.62% of deaths associated with resistance) (Figure 2). For the indicator of DALY, *Escherichia coli* and *Klebsiella pneumoniae* were also the two major pathogens responsible for the AMR-related deaths in almost all 21 GBD regions (Figure S3).

### 3.3. Antibiotic-Specific Burden of Bacterial AMR in UTI

Globally, four antibiotic classes were each responsible for more than 100 thousand deaths associated with AMR: fluoroquinolones (169.15 thousand [95% UI: 119.64–240.75]), third-generation cephalosporins (113.06 thousand [95% UI: 78.60–163.97]), aminopenicillin (107.72 thousand [95% UI: 84.82–139.29]), and β-lactam or β-lactamase inhibitors (104.54 thousand [95% UI: 76.74–144.36]). For deaths attributed to AMR, fluoroquinolones (21.12 thousand [95% UI: 14.14–31.53]) were still responsible for the most deaths in 2019, followed by carbapenems (21.12 thousand [95% UI: 14.14–31.53]) and third-generation cephalosporins (10.98 thousand [95% UI: 6.15–18.12]) (Figure 1C). The DALYs caused by 13 antibiotic classes were shown in Figure S4.

**Figure 3:**
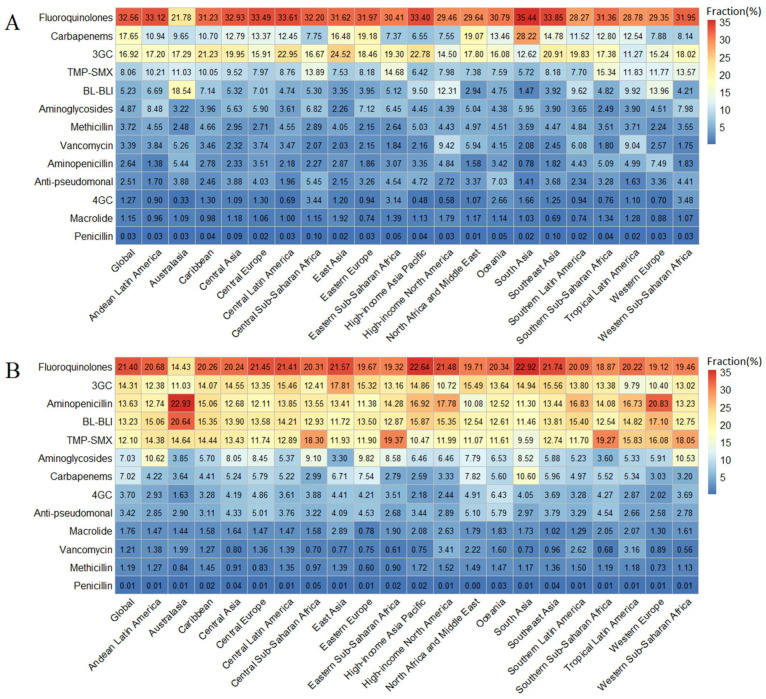
Fractions of deaths attributable to (**A**) and associated with (**B**) bacterial AMR among 21 GBD regions and 13 antibiotic classes, 2019. 3GC = third-generation cephalosporins. 4GC = fourth-generation cephalosporins. Anti-pseudomonal = anti-pseudomonal penicillin or beta-lactamase inhibitors. BL-BLI = β-lactam or β-lactamase inhibitors. Resistance to 1+ = resistance to one or more drugs. TMP-SMX = trimethoprim/sulfamethoxazole. AMR = antimicrobial resistance. GBD = Global Burden of Diseases, Injuries, and Risk Factors Study.

Globally and in almost all 21 GBD regions, fluoroquinolones, carbapenems, and third-generation cephalosporins were the top three leading antibiotics attributed to AMR (Figure 3), but there were also a few exceptions. For instance, in Australasia, the second-ranked antibiotic class was β-lactam or β-lactamase inhibitors. For deaths associated with AMR, fluoroquinolones, third-generation cephalosporins, aminopenicillin, β-lactam or β-lactamase inhibitors, and trimethoprim/sulfamethoxazole were the top five antibiotics causing deaths. In Australasia and Western Europe, aminopenicillin ranked first, and in eastern and southern Sub-Saharan Africa, trimethoprim/sulfamethoxazole ranked first. The percentages of AMR burden caused by the same antibiotic class also differed substantially across GBD super-regions. For example, the percentages of AMR death caused by fluoroquinolones ranged from the highest in South Asia (35.44% of deaths attributable to resistance and 22.92% of deaths associated with resistance) to the lowest in Australasia (21.78% of deaths attributable to resistance and 14.43% of deaths associated with resistance) (Figure 3). The major antibiotic classes causing DALYs attributed to and associated with AMR were similar to those for the death index (Figure S5).

### 3.4. Pathogen-Antibiotic Specific Burden of Bacterial AMR in UTI

**Figure 4:**
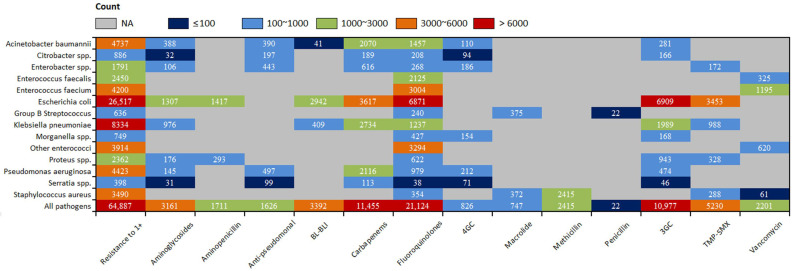
All-age count of deaths attributable to bacterial AMR globally among 13 antibiotic classes and 14 pathogens, 2019. The deaths associated with resistance are not shown due to the very high levels of correlation for resistance patterns between some drugs. 3GC = third-generation cephalosporins. 4GC = fourth-generation cephalosporins. Anti-pseudomonal = anti-pseudomonal penicillin or beta-lactamase inhibitors. BL-BLI = β-lactam or β-lactamase inhibitors. NA = not applicable. Resistance to 1+ = resistance to one or more drugs. TMP-SMX = trimethoprim/sulfamethoxazole. AMR = antimicrobial resistance. Error bars indicate 95% uncertainty intervals.

Among the 66 combinations, the resistance-attributable death caused by 2 pathogen-drug combinations exceeded 6000 in 2019 globally: third-generation cephalosporin-resistant *Escherichia coli* (6909 deaths) and fluoroquinolone-resistant *Escherichia coli* (6871 deaths). Four pathogen-drug combinations each caused between 3000 and 6000 resistance-attributable deaths: carbapenem-resistant *Escherichia coli* (3617 deaths), trimethoprim/sulfamethoxazole -resistant *Escherichia coli* (3453 deaths), fluoroquinolone-resistant other *enterococci* (3294 deaths), and fluoroquinolone-resistant *Enterococcus faecium* (3004 deaths) (Figure 4). For the combination of third-generation cephalosporin-resistant *Escherichia coli*, it caused the most deaths in South Asia (2296 deaths) among 21 GBD regions, and for the combination of fluoroquinolone-resistant *Escherichia coli*, it caused the most deaths in South Asia (2296 deaths) and East Asia (1054 deaths) among 21 GBD regions (Table S2).

## 4. Discussion

Globally, 64.89 thousand deaths from AMR in UTI would be prevented if all drug-resistant UTIs were replaced by susceptible infections, and 0.26 million deaths from AMR in UTI would be avoided if all drug-resistant UTIs were replaced by non-infections in 2019. Based on the counterfactual scenario of susceptive infections, the number of deaths caused by AMR in UTI was almost the same as the sum of deaths caused by testicular cancer, urolithiasis, and other uncommon urology diseases in 2019 [13]. Based on the counterfactual scenario of non-infection, AMR in UTI was the fourth most infectious syndrome leading to AMR-related cause of death globally, just behind lower respiratory infections and all related infections in the thorax, bloodstream infections, and peritoneal and intra-abdominal infections [4]. Additionally, the disease burden of UTI is increasing [9], and antibiotics are usually recommended for the treatment of UTI [14,15,16] and as prophylactics, such as during prostate biopsy [17] and urological surgery [18]. Considering these facts, AMR in UTI is a health problem that cannot be ignored, both for the management of urology disease and for global antibiotic resistance. Compared with studies which reported AMR in UTI in selected countries or regions [10,11,12], this study provided global, location-specific, pathogen-specific, and antibiotic-specific AMR-related burdens. The estimates presented in this study can be compared directly among different regions, pathogens, and antibiotics, providing valuable information for the management of AMR in urology.

The all-age death rates of AMR in UTI were high in some areas of Latin America and Europe and lowest in areas of sub-Saharan Africa. These findings differ from the results in terms of the AMR burden for all infectious syndromes, in which the highest death rates occurred in the four sub-Saharan Africa areas [4]. The burden of bacterial AMR in UTI in a region was consistent with the burden of UTI in the corresponding region [9]. Additionally, socioeconomic level, such as infrastructure construction and government supervision, might influence AMR [19]. For example, in addition to the relatively low UTI burden [9], the other reasons for the lowest bacterial AMR burden in UTI in sub-Saharan African might include the scarcity of laboratory infrastructure resulting in the lack of adequate quality of data [20] and insufficient government regulations resulting in the easy acquisition of antibiotics without prescription [21] or counterfeited medications [22]. As the patterns and drivers of bacterial AMR differ among regions, local data provide useful information for policy-making, and special tailored strategies should be developed considering local socioeconomic factors.

*Escherichia coli* and *Klebsiella pneumoniae* dominated the deaths attributed to and associated with AMR in UTI. This result was consistent with the results reported in other studies [10,19,20]. When considering the antibiotic classes of drug, fluoroquinolones, carbapenems, third-generation cephalosporins, aminopenicillin, β-lactam or β-lactamase inhibitors, and trimethoprim/sulfamethoxazole were the most common antibiotics causing death. However, different from the consistency of major pathogens in most regions worldwide, the variation in leading antibiotics causing death was great among different regions, although fluoroquinolones were the major antibiotic class in most regions. For instance, in terms of deaths attributable to AMR, the second-ranked antibiotic class in Australasia was β-lactam or β-lactamase inhibitors, and the third-ranked antibiotic class was trimethoprim/sulfamethoxazole in four sub-Saharan Africa areas (central, eastern, southern, and western). This discrepancy may be caused by variations in the availability and affordability of different antibiotics in regions with different levels of economic development [23]. Moreover, other factors such as the awareness and knowledge of appropriate use of antimicrobials, government regulation, and sanitation and hygiene might also influence the use and resistance of antibiotics in one region [2].

The regional data about bacteria-antibiotic combinations causing AMR-attributed deaths is helpful for figuring out the most serious category of AMR in UTI and for selecting appropriate treatments in that region. For example, in South Asia and East Asia, third-generation cephalosporin-resistant *Escherichia coli* and fluoroquinolone-resistant *Escherichia coli* are the two combinations causing the most deaths from UTI. Targeting measures should be employed to treat UTI caused by *Escherichia coli* using appropriate antibiotics. Noteworthily, *Klebsiella pneumoniae* was the second-leading urinary pathogen responsible for AMR-related deaths, yet the number of deaths caused by *Klebsiella pneumoniae* in combination with any antibiotic class was not high among all 66 bacteria-antibiotic combinations. In 2017, the WHO published a priority list of antibiotic-resistant pathogens that pose the greatest threat to human health to help in prioritizing the research and development of new and effective antibiotic treatments [24]. Among the six bacteria-antibiotic combinations which caused more than 3000 AMR attributed deaths estimated in this study, only carbapenem-resistant *Escherichia coli* and third-generation cephalosporin-resistant *Escherichia coli* were included in the list, and these two combinations were identified with a critical priority, which is the highest priority in the list. However, fluoroquinolone-resistant *Escherichia coli*, the second-leading pathogen-antibiotic combination causing AMR-attributed death, was not included. Some countries also issued bacteria priority lists, such as the USA [25] and Canada [26]. These lists included some different bacteria as they were developed based on global or national public health priorities, but they were effective in increasing scientific, political, and public awareness of antibiotic resistance. To our knowledge, as this study is the first to report AMR-related burden in urology, the estimates presented by this study can provide valuable information for prioritizing pathogen-antibiotic combinations in urology.

To tackle the challenge of bacterial AMR, global actions have been taken such as the Global Action Plan on AMR [27] and the One Health Global Leaders Group on AMR [28]. The burden of AMR in UTI is the result of a series of factors, such as the high incidence of UTI, the extensive and inappropriate use of antibiotics, the scarcity of laboratory infrastructure, and health and economic development levels. Therefore, it is obvious that the development and implementation of locational or national measures and policies are of great importance. Considering the vast consensus about the adoption of empirical antibiotic treatment for UTI, antibiotic resistance surveillance is essential for informing and monitoring the impacts of local, national, and global strategies to optimize the use of these drugs [29]. There have been substantial national and international efforts aimed at strengthening AMR surveillance, including the Global Antimicrobial Resistance and Use Surveillance System (GLASS) [30] and the Central Asian and European Surveillance of Antimicrobial Resistance (CAESAR) [31]. However, surveillance activities in low- and middle-income countries have been relatively slow paced [32]. Meanwhile, laboratory capacity in these regions, including the capacity for laboratory data management, need to be improved [32,33].

Another important aspect of controlling antibiotic resistance in UTI is the rational use of antibiotics. Antibiotic treatment is the basic component for UTI therapy [15] and is frequently performed empirically [34]. In empirical treatment, the bacteria might not be sensitive to the antibiotic administered, resulting in treatment failure, and in some cases, physicians tend to prescribe broad spectrum and ‘last resort’ antibiotics to minimize treatment failure, which in turn increases the probability of antibiotic resistance. For example, a cross-sectional study conducted in nursing homes in the USA found that half of the antibiotic prescriptions for a suspected UTI in residents without catheters occurred with no documented signs or symptoms of a UTI [35]. However, the guideline did not recommend treating asymptomatic bacteriuria in elderly institutionalized patients [15]. In addition to antibiotic treatment, nonantibiotic prevention and treatment options, such as probiotics, nonsteroidal anti-inflammatory drugs, and immunotherapy, can be included in the therapeutic strategy to reduce the likelihood of AMR.

Several limitations should be acknowledged. First, although GBD collaborations made extensive efforts to obtain data from multiple sources, data about AMR are sparse in some low-income and middle-income countries. To tackle this problem, several methodological assumptions were made by researchers, and the fits of modeled estimates with available data were validated. Second, as there exists a discrepancy in the diagnostic standards, detection methods, and supervision systems among different regions and countries, the quality of the data for different sources is uneven. Third, data on the UTI-related AMR burden of different age groups, genders, and infection sites are unavailable. UTIs such as lower urinary tract infections often occur in elders and females, data on the burden of UTI-related AMR in these high-risk populations at different infection sites can help in taking measures to control antibiotic resistance.

## 5. Conclusions

Bacterial AMR in UTI is a health problem that cannot be overlooked in urology, especially in southern Latin America, tropical Latin America and Europe. *Escherichia coli* and *Klebsiella pneumoniae* play the dominant roles in AMR, and resistance is high among multiple classes of antibiotic including fluoroquinolones, carbapenems, and third-generation cephalosporins. Special tailored strategies including enhanced surveillance and rational use of antibiotics should be developed for different regions according to the region-specific pathogen-antibiotic situations and resources. 

## Figures and Tables

**Table 1:** Deaths, DALYs, YLDs, and YLLs (in counts and all-age rates) associated with and attributable to bacterial AMR in UTI, globally and by GBD super-region, 2019.

	Associated with Resistance				Attributable to Resistance			
	Deaths	DALYs	YLDs	YLLs	Deaths	DALYs	YLDs	YLLs
**Counts, thousands**								
Global	255.802 (182.553–363.908)	5607.970 (4081.637–7991.093)	167.469 (100.767–256.144)	5440.501 (3910.668–7813.322)	64.887 (45.859–94.345)	1438.848 (1020.287–2101.201)	33.512 (19.367–52.959)	1405.336 (992.093–2061.045)
Central Europe, eastern Europe, and central Asia	23.891 (16.045–35.879)	516.223 (358.717–757.066)	12.766 (7.650–19.945)	503.457 (348.368–743.746)	6.048 (4.001–9.321)	130.323 (88.640–196.693)	2.534 (1.442–4.126)	127.789 (86.409–193.407)
High income	53.856 (41.957–69.587)	732.320 (556.679–995.742)	25.261 (15.378–37.566)	707.059 (527.611–971.935)	12.361 (9.453–16.619)	168.975 (125.217–240.207)	4.573 (2.729–7.012)	164.402 (120.670–234.943)
Latin America and Caribbean	31.191 (24.059–40.852)	614.994 (470.752–822.682)	25.018 (15.092–38.827)	589.975 (448.038–793.591)	7.498 (5.584–10.170)	148.274 (110.323–204.156)	4.780 (2.789–7.585)	143.494 (105.170–199.304)
North Africa and Middle East	10.056 (5.100–17.225)	243.098 (124.193–430.123)	12.452 (7.259–20.035)	230.645 (111.096–414.670)	2.710 (1.365–4.784)	65.167 (32.552–117.214)	2.623 (1.455–4.473)	62.544 (29.914–114.777)
South Asia	72.124 (53.057–96.020)	1953.222 (1487.744–2542.502)	58.569 (33.299–92.863)	1894.653 (1426.036–2486.039)	20.067 (14.564–27.121)	540.202 (396.373–722.421)	12.534 (6.778–20.872)	527.667 (385.580–706.749)
Southeast Asia, east Asia, and Oceania	53.400 (30.379–88.532)	1177.714 (689.684–1939.853)	17.028 (9.800–27.123)	1160.687 (676.277–1915.141)	13.529 (7.563–22.868)	298.833 (170.977–499.896)	3.399 (1.815–5.750)	295.434 (168.241–495.854)
Sub-Saharan Africa	11.303 (6.960–15.950)	370.421 (243.293–519.435)	16.385 (8.521–29.816)	354.036 (228.904–501.817)	2.677 (1.663–3.798)	87.042 (57.826–122.462)	3.069 (1.531–5.798)	83.974 (55.246–119.396)
**ASR rates, per 100,000**								
Global	3.310 (2.360–4.700)	72.478 (52.752–103.278)	2.160 (1.300–3.310)	70.314 (50.542–100.980)	0.839 (0.593–1.220)	18.596 (13.186–27.156)	0.433 (0.250–0.684)	18.163 (12.822–26.637)
Central Europe, eastern Europe, and central Asia	5.720 (3.840–8.590)	123.580 (85.874–181.236)	3.060 (1.830–4.770)	120.523 (83.397–178.047)	1.450 (0.958–2.230)	31.198 (21.220–47.087)	0.607 (0.345–0.988)	30.592 (20.686–46.300)
High income	4.970 (3.870–6.420)	67.559 (51.355–91.860)	2.330 (1.420–3.470)	65.228 (48.674–89.664)	1.140 (0.872–1.530)	15.588 (11.552–22.160)	0.422 (0.252–0.647)	15.166 (11.132–21.674)
Latin America and Caribbean	5.340 (4.120–6.990)	105.239 (80.556–140.779)	4.280 (2.580–6.640)	100.958 (76.669–135.801)	1.280 (0.955–1.740)	25.373 (18.879–34.935)	0.818 (0.477–1.300)	24.555 (17.997–34.105)
North Africa and Middle East	1.650 (0.838–2.830)	39.936 (20.403–70.661)	2.050 (1.190–3.290)	37.891 (18.251–68.122)	0.445 (0.224–0.786)	10.706 (5.350–19.256)	0.431 (0.239–0.735)	10.275 (4.910–18.856)
South Asia	4.000 (2.940–5.320)	108.200 (82.414–140.843)	3.240 (1.840–5.140)	104.955 (78.996–137.715)	1.110 (0.807–1.500)	29.925 (21.957–40.019)	0.694 (0.375–1.160)	29.230 (21.359–39.151)
Southeast Asia, east Asia, and Oceania	2.470 (1.410–4.100)	54.542 (31.941–89.839)	0.789 (0.454–1.260)	53.754 (31.320–88.694)	0.627 (0.350–1.060)	13.840 (7.920–23.151)	0.157 (0.084–0.266)	13.682 (7.790–22.964)
Sub-Saharan Africa	1.050 (0.645–1.480)	34.355 (22.565–48.176)	1.520 (0.790–2.770)	32.836 (21.230–46.542)	0.248 (0.154–0.352)	8.070 (5.360–11.358)	0.285 (0.142–0.538)	7.790 (5.120–11.074)

DALYs = disability-adjusted life-years. YLD = year lived with disability. YLL = years of life lost. AMR = antimicrobial resistance. GBD = Global Burden of Diseases, Injuries, and Risk Factors Study. ASR = age standardized rate.

## Data Availability

Publicly available datasets were analyzed in this study. This data can be found here: https://ghdx.healthdata.org/record/ihme-data/global-bacterial-antimicrobial-resistance-burden-estimates-2019 (accessed on 22 February 2022).

## Supplementary Materials

The following supporting information can be downloaded at: https://www.mdpi.com/article/10.3390/jcm11102817/s1. Table S1: List of GBD location hierarchy by region. Table S2: All-age count of deaths attributable to bacterial AMR among 13 antibiotic classes and 14 pathogens for 21 GBD regions. Figure S1: All-age rate of DALYs attributable to and associated with bacterial AMR among 21 GBD regions, 2019. Figure S2: All-age count of DALYs attributable to and associated with bacterial AMR globally among 14 pathogens, 2019. Figure S3: Fraction of DALYs attributable to (A) and associated with (B) bacterial AMR among 21 GBD regions and 14 pathogens, 2019. Figure S4: All-age count of DALYs attributable to and associated with bacterial AMR globally among 13 antibiotic classes, 2019. Figure S5: Fraction of DALYs attributable to (A) and associated with (B) bacterial AMR among 21 GBD regions and 13 antibiotic classes, 2019.

## References

[1] Hernando-Amado S., Coque T.M., Baquero F., Martínez J.L. (2019). Defining and combating antibiotic resistance from One Health and Global Health perspectives. Nat. Microbiol..

[2] World Health Organization Antimicrobial Resistance. https://www.who.int/news-room/fact-sheets/detail/antimicrobial-resistance.

[3] Laxminarayan R., Duse A., Wattal C., Zaidi A.K., Wertheim H.F., Sumpradit N., Vlieghe E., Hara G.L., Gould I.M., Goossens H. (2013). Antibiotic resistance-the need for global solutions. Lancet Infect. Dis..

[4] Antimicrobial Resistance Collaborators (2022). Global burden of bacterial antimicrobial resistance in 2019: A systematic analysis. Lancet.

[5] Laxminarayan R. (2022). The overlooked pandemic of antimicrobial resistance. Lancet.

[6] Wagenlehner F.M.E., Johansen T.E.B., Cai T., Koves B., Kranz J., Pilatz A., Tandogdu Z. (2020). Epidemiology, definition and treatment of complicated urinary tract infections. Nat. Rev. Urol..

[7] Flores-Mireles A.L., Walker J.N., Caparon M., Hultgren S.J. (2015). Urinary tract infections: Epidemiology, mechanisms of infection and treatment options. Nat. Rev. Microbiol..

[8] Palacios-Ceña D., Florencio L.L., Hernández-Barrera V., Fernandez-de-Las-Peñas C., de Miguel-Diez J., Martínez-Hernández D., Carabantes-Alarcón D., Jimenez-García R., Lopez-de-Andres A., Lopez-Herranz M. (2021). Trends in Incidence and Outcomes of Hospitalizations for Urinary Tract Infection among Older People in Spain (2001–2018). J. Clin. Med..

[9] Zhu C., Wang D.Q., Zi H., Huang Q., Gu J.M., Li L.Y., Guo X.P., Li F., Fang C., Li X.D. (2021). Epidemiological trends of urinary tract infections, urolithiasis and benign prostatic hyperplasia in 203 countries and territories from 1990 to 2019. Mil. Med. Res..

[10] Tandogdu Z., Cek M., Wagenlehner F., Naber K., Tenke P., van Ostrum E., Johansen T.B. (2014). Resistance patterns of nosocomial urinary tract infections in urology departments: 8-year results of the global prevalence of infections in urology study. World J. Urol..

[11] Choe H.S., Lee S.J., Cho Y.H., Çek M., Tandoğdu Z., Wagenlehner F., Bjerklund-Johansen T.E., Naber K. (2018). Aspects of urinary tract infections and antimicrobial resistance in hospitalized urology patients in Asia: 10-Year results of the Global Prevalence Study of Infections in Urology (GPIU). J. Infect. Chemother..

[12] Zilberberg M.D., Nathanson B.H., Sulham K., Shorr A.F. (2021). Multiple antimicrobial resistance and outcomes among hospitalized patients with complicated urinary tract infections in the US, 2013–2018: A retrospective cohort study. BMC Infect. Dis..

[13] GBD 2019 Diseases and Injuries Collaborators (2020). Global burden of 369 diseases and injuries in 204 countries and territories, 1990–2019: A systematic analysis for the Global Burden of Disease Study 2019. Lancet.

[14] Anger J., Lee U., Ackerman A.L., Chou R., Chughtai B., Clemens J.Q., Hickling D., Kapoor A., Kenton K.S., Kaufman M.R. (2019). Recurrent Uncomplicated Urinary Tract Infections in Women: AUA/CUA/SUFU Guideline. J. Urol..

[15] Bonkat G., Bartoletti R., Bruyère F., Cai T., Geerlings S.E., Köves B., Schubert S., Wagenlehner F. EAU Guidelines on Urological Infections 2020. https://uroweb.org/wp-content/uploads/EAU-Guidelines-on-Urological-infections-2020.pdf.

[16] Fox M.T., Amoah J., Hsu A.J., Herzke C.A., Gerber J.S., Tamma P.D. (2020). Comparative Effectiveness of Antibiotic Treatment Duration in Children with Pyelonephritis. JAMA Netw. Open.

[17] Mottet N., Cornford P., van den Bergh R.C.N., Briers E., de Santis M., Fanti S., Gillessen S., Grummet J., Henry A.M., Lam T.B. EAU EANM ESTRO ESUR SIOG Guidelines on Prostate Cancer 2020. https://uroweb.org/wp-content/uploads/EAU-EANM-ESTRO-ESUR-SIOG-Guidelines-on-Prostate-Cancer-2020v4-1.pdf.

[18] Bruyere F., Pilatz A., Boehm A., Pradere B., Wagenlehner F., Vallee M. (2020). Associated measures to antibiotic prophylaxis in urology. World J. Urol..

[19] Esposito S., Maglietta G., di Costanzo M., Ceccoli M., Vergine G., la Scola C., Malaventura C., Falcioni A., Iacono A., Crisafi A. (2021). Retrospective 8-Year Study on the Antibiotic Resistance of Uropathogens in Children Hospitalised for Urinary Tract Infection in the Emilia-Romagna Region, Italy. Antibiotics.

[20] Huang L., Huang C., Yan Y., Sun L., Li H. (2021). Urinary Tract Infection Etiological Profiles and Antibiotic Resistance Patterns Varied Among Different Age Categories: A Retrospective Study from a Tertiary General Hospital During a 12-Year Period. Front. Microbiol..

[21] Sakeena M.H.F., Bennett A.A., McLachlan A.J. (2018). Non-prescription sales of antimicrobial agents at community pharmacies in developing countries: A systematic review. Int. J. Antimicrob. Agents.

[22] Kelesidis T., Falagas M.E. (2015). Substandard/counterfeit antimicrobial drugs. Clin. Microbiol. Rev..

[23] Monnier A.A., Schouten J., Tebano G., Zanichelli V., Huttner B.D., Pulcini C., Årdal C., Harbarth S., Hulscher M.E., Gyssens I.C. (2019). Ensuring Antibiotic Development, Equitable Availability, and Responsible Use of Effective Antibiotics: Recommendations for Multisectoral Action. Clin. Infect. Dis..

[24] World Health Organization WHO Publishes List of Bacteria for Which New Antibiotics Are Urgently Needed. https://www.who.int/news/item/27-02-2017-who-publishes-list-of-bacteria-for-which-new-antibiotics-are-urgently-needed.

[25] Centers for Disease Control and Prevention (2019). Antibiotic Resistance Threats in the United States. https://www.cdc.gov/drugresistance/pdf/threats-report/2019-ar-threats-report-508.pdf.

[26] Health Canada Notice—Health Canada’s update to the Pathogens of Interest List and Ongoing Efforts to Support Innovative Human Therapeutic Products to Combat Antimicrobial Resistance (AMR). https://www.canada.ca/en/health-canada/programs/consultation-new-addition-pathogens-interest-list/document.html#a4.

[27] World Health Organization (2016). Global Action Plan on Antimicrobial Resistance.

[28] Zarocostas J. (2020). New international group on antimicrobial resistance. Lancet.

[29] Falcone A., Amantea D., Levato A., Arone F., Morrone L.A., Bagetta D., Florio L., Lista M.R., Bagetta G. (2006). Outcomes of a pharmacoepidemiological survey on the antibiotic treatment of uncomplicated acute cystitis in community. Pharmacol. Res..

[30] World Health Organization Global Antimicrobial Resistance and Use Surveillance System (GLASS). https://www.who.int/initiatives/glass.

[31] World Health Organization Central Asian and European Surveillance of Antimicrobial Resistance (CAESAR). https://www.euro.who.int/en/health-topics/disease-prevention/antimicrobial-resistance/surveillance/central-asian-and-european-surveillance-of-antimicrobial-resistance-caesar.

[32] Frost I., Kapoor G., Craig J., Liu D., Laxminarayan R. (2021). Status, challenges and gaps in antimicrobial resistance surveillance around the world. J. Glob. Antimicrob. Resist..

[33] Turner P., Rupali P., Opintan J.A., Jaoko W., Feasey N.A., Peacock S.J., Ashley E.A. (2021). Laboratory informatics capacity for effective antimicrobial resistance surveillance in resource-limited settings. Lancet Infect. Dis..

[34] Wagenlehner F.M., Naber K.G. (2019). Understanding clinical variables to improve empirical antibiotic therapy for UTI. Nat. Rev. Urol..

[35] Phillips C.D., Adepoju O., Stone N., Moudouni D.K., Nwaiwu O., Zhao H., Frentzel E., Mehr D., Garfinkel S. (2012). Asymptomatic bacteriuria, antibiotic use, and suspected urinary tract infections in four nursing homes. BMC Geriatr..

