# Supplementary material for: Global and Regional Burden of Bacterial Antimicrobial Resistance in Urinary Tract Infections in 2019
Source: J Clin Med. 2022 May 17;11(10):2817. doi: 10.3390/jcm11102817 (PMC9147874; doi:10.3390/jcm11102817)
Supplement: Supplementary file 1 [file jcm-11-02817-s001.zip › jcm-1654425-supplementary.pdf]

Supplemental Material

Table S1 List of GBD location hierarchy by region.

Table S2 All-age count of deaths attributable to bacterial AMR among 13 antibiotic classes and 14 pathogens for 21 GBD regions.

Figure S1 All-age rate of DALYs attributable to and associated with bacterial AMR among 21 GBD regions, 2019.

Figure S2 All-age count of DALYs attributable to and associated with bacterial AMR globally among 14 pathogens, 2019.

Figure S3 Fraction of DALYs attributable to (A) and associated with (B) bacterial AMR among 21 GBD regions and 14 pathogens, 2019.

Figure S4 All-age count of DALYs attributable to and associated with bacterial AMR globally among 13 antibiotic classes, 2019.

Figure S5 Fraction of DALYs attributable to (A) and associated with (B) bacterial AMR among 21 GBD regions and 13 antibiotic classes, 2019.

Table S1. List of GBD location hierarchy by region.

GBD super-region	GBD region	Country
Central Europe, eastern Europe, and central Asia	Central Asia	Armenia
		Azerbaijan
		Georgia
		Kazakhstan
		Kyrgyzstan
		Mongolia
		Tajikistan
		Turkmenistan
		Uzbekistan
	Central Europe	Albania
		Bosnia and Herzegovina
		Bulgaria
		Croatia
		Czech
		Hungary
		Montenegro
		North Macedonia
		Poland
		Romania
		Serbia
		Slovakia
		Slovenia
	Eastern Europe	Belarus
		Estonia
		Latvia
		Lithuania
		Moldova
		Russia
		Ukraine
High income	Australasia	Australia
		New Zealand
	High-income Asia Pacific	Brunei
		Japan
		Singapore
		South Korea
	High-income North America	Canada
		Greenland
		USA
	Southern Latin America	Argentina
		Chile

		Uruguay
	Western Europe	Andorra
		Austria
		Belgium
		Cyprus
		Denmark
		Finland
		France
		Germany
		Greece
		Iceland
		Ireland
		Israel
		Italy
		Luxembourg
		Malta
		Monaco
		Netherlands
		Norway
		Portugal
		San Marino
		Spain
		Sweden
		Switzerland
		UK
Latin America and Caribbean	Andean Latin America	Bolivia
		Ecuador
		Peru
	Caribbean	Antigua and Barbuda
		The Bahamas
		Barbados
		Belize
		Bermuda
		Cuba
		Dominica
		Dominican Republic
		Grenada
		Guyana
		Haiti
		Jamaica
		Puerto Rico
		Saint Kitts and Nevis

		Saint Lucia
		Saint Vincent and the Grenadines
		Suriname
		Trinidad and Tobago
		Virgin Islands
	Central Latin America	Colombia
		Costa Rica
		El Salvador
		Guatemala
		Honduras
		Mexico
		Nicaragua
		Panama
		Venezuela
	Tropical Latin America	Brazil
		Paraguay
North Africa and Middle East	North Africa and Middle East	Afghanistan
		Algeria
		Bahrain
		Egypt
		Iran
		Iraq
		Jordan
		Kuwait
		Lebanon
		Libya
		Morocco
		Oman
		Palestine
		Qatar
		Saudi Arabia
		Sudan
		Syria
		Tunisia
		Turkey
		United Arab Emirates
		Yemen
South Asia	South Asia	Bangladesh
		Bhutan
		India
		Nepal

		Pakistan
Southeast Asia, east Asia, and Oceania	East Asia	China
		North Korea
		Taiwan (province of China)
	Oceania	American Samoa
		Cook Islands
		Fiji
		Guam
		Kiribati
		Marshall Islands
		Federated States of Micronesia
		Nauru
		Niue
		Northern Mariana Islands
		Palau
		Papua New Guinea
		Samoa
		Solomon Islands
		Tokelau
		Tonga
		Tuvalu
		Vanuatu
	Southeast Asia	Cambodia
		Indonesia
		Laos
		Malaysia
		Maldives
		Mauritius
		Myanmar
		Philippines
		Seychelles
		Sri Lanka
		Thailand
		Timor-Leste
		Vietnam
Sub-Saharan Africa	Central sub-Saharan Africa	Angola
		Central African Republic
		Congo (Brazzaville)
		DR Congo
		Equatorial Guinea
		Gabon

		Burundi
		Comoros
		Djibouti
		Eritrea
		Ethiopia
		Kenya
		Madagascar
		Malawi
		Mozambique
		Rwanda
		Somalia
		South Sudan
		Uganda
		Tanzania
		Zambia
	Southern sub-Saharan Africa	Botswana
		Eswatini
		Lesotho
		Namibia
		South Africa
		Zimbabwe
	Western sub-Saharan Africa	Benin
		Burkina Faso
		Cape Verde
		Cameroon
		Chad
		Côte d'Ivoire
		The Gambia
		Ghana
		Guinea
		Guinea-Bissau
		Liberia
		Mali
		Mauritania
		Niger
		Nigeria
		São Tomé and Príncipe
		Senegal
		Sierra Leone
		Togo

Table S2. All-age count of deaths attributable to bacterial AMR among 13 antibiotic classes and 14 pathogens for 21 GBD regions.

Pathogen-drug combinations	GBD regions																				
	Global	Andean Latin America	Australasia	Caribbean	Central Asia	Central Europe	Central Latin America	Central Sub-Saharan Africa	East Asia	Eastern Europe	Eastern Sub-Saharan Africa	High-income Asia Pacific	High-income North America	North Africa and Middle East	Oceania	South Asia	Southeast Asia	Southern Latin America	Southern Sub-Saharan Africa	Tropical Latin America	Western Europe	Western Sub-Saharan Africa
All pathogens-Aminoglycosides	3161.18	46.42	5.83	14.67	53.91	84.97	96.91	20.04	172.57	259.95	62.97	78.80	178.80	136.72	3.14	1193.92	226.81	46.63	6.39	151.42	228.26	91.73
All pathogens-Aminopenicillin	1710.61	7.53	9.85	10.27	22.29	50.54	58.49	6.66	219.83	67.73	30.00	59.25	197.33	42.95	2.45	156.29	105.67	56.60	13.08	193.94	378.86	21.08
All pathogens-Anti-pseudomonal	1626.41	9.32	7.02	9.11	37.15	58.12	52.54	16.00	164.33	118.87	44.33	83.56	111.01	91.47	5.04	282.80	213.80	29.93	8.44	63.18	169.98	50.66
All pathogens-BL-BLI	3392.15	36.60	33.56	26.43	50.97	100.98	127.22	15.56	256.22	144.23	50.02	168.18	501.92	79.66	3.41	295.26	227.67	122.78	12.40	385.20	705.83	48.35
All pathogens-Carbapenems	11454.58	59.91	17.47	39.58	122.54	192.68	334.54	22.77	1260.06	700.09	71.92	115.99	307.93	516.83	9.65	5662.08	859.57	147.06	32.91	486.82	398.51	93.56
All pathogens-Fluoroquinolones	21124.27	181.27	39.43	115.55	315.47	482.61	903.01	94.60	2418.53	1166.79	296.75	591.58	1200.69	803.34	22.08	7112.57	1968.21	360.90	80.64	1117.83	1483.78	367.41
All pathogens-4GC	825.98	4.94	0.59	4.81	10.49	18.74	18.56	10.12	91.66	34.23	30.61	8.46	23.52	28.94	1.91	333.87	72.58	11.98	1.95	42.79	35.16	40.06
All pathogens-Macrolide	746.55	5.27	1.96	3.61	11.35	15.23	26.95	3.38	146.91	27.08	13.54	20.04	72.95	31.81	0.82	205.79	40.36	9.45	3.45	49.58	44.59	12.35
All pathogens-Methicillin	2415.20	24.93	4.48	17.25	28.25	39.08	122.32	8.50	309.45	78.54	25.77	89.16	180.62	134.81	3.24	719.92	259.87	61.82	9.03	143.94	113.40	40.87
All pathogens-Penicillin	21.68	0.17	0.06	0.16	0.90	0.28	0.69	0.29	1.45	1.19	0.48	0.76	1.12	0.38	0.04	4.99	5.79	0.26	0.09	0.86	1.30	0.40
All pathogens-Resistance to 1+	64886.51	547.38	181.03	369.96	957.89	1441.27	2686.42	293.80	7648.03	3649.29	975.98	1771.18	4076.04	2710.39	71.72	20066.57	5814.33	1276.52	257.14	3883.53	5055.63	1149.81
All pathogens-3GC	10977.17	94.16	31.29	78.53	191.10	229.26	616.59	48.99	1875.17	673.74	188.37	403.42	591.10	482.38	11.53	2532.99	1215.65	253.16	44.69	437.65	770.54	207.19
All pathogens-TMP-SMX	5229.68	55.86	19.96	37.18	91.23	114.91	235.39	40.80	576.25	298.40	143.25	113.79	325.24	200.04	5.44	1148.08	475.63	98.30	39.44	459.35	595.28	156.06
All pathogens-Vancomycin	2201.05	20.99	9.52	12.80	22.24	53.87	93.22	6.08	155.60	78.46	17.96	38.18	383.81	161.07	2.98	418.01	142.72	77.66	4.62	350.94	130.13	20.09
Acinetobacter baumannii-Aminoglycosides	387.81	3.56	0.29	2.11	9.35	12.01	23.01	0.90	46.39	33.02	2.62	6.77	13.52	37.37	0.66	111.36	45.81	5.25	2.12	16.45	10.60	4.67
Acinetobacter baumannii-Anti-pseudomonal	390.18	1.27	2.35	2.33	11.42	12.25	3.48	7.11	18.90	18.41	18.73	10.22	16.44	28.13	3.14	52.21	113.10	1.11	4.92	5.40	35.26	24.24
Acinetobacter baumannii-BL-BLI	41.23	0.00	0.06	0.17	0.03	1.76	0.43	0.01	0.92	0.14	0.33	10.06	1.84	0.74	0.07	1.90	4.38	0.01	0.00	1.63	16.76	0.00
Acinetobacter baumannii-Carbapenems	2070.09	17.94	3.07	10.41	37.86	55.48	106.25	3.58	548.74	126.74	9.45	29.87	79.60	147.94	3.07	493.22	210.89	30.30	11.45	81.47	49.40	13.27

Acinetobacter baumannii-Fluoroquinolones	1457.24	12.79	1.32	8.47	33.19	46.69	75.75	4.09	239.62	107.91	11.62	16.63	56.27	132.20	2.56	408.68	152.99	21.93	11.32	64.55	37.15	11.55
Acinetobacter baumannii-4GC	109.79	0.43	0.15	0.78	0.14	2.45	1.40	0.00	55.76	1.09	0.30	1.77	4.12	1.43	0.28	15.95	10.99	0.43	0.03	2.56	9.72	0.00
Acinetobacter baumannii-Resistance to 1+	4737.39	36.83	10.12	25.23	92.97	134.11	218.59	16.06	972.23	291.28	46.16	98.45	220.78	351.15	10.08	1107.86	560.06	60.99	30.96	179.98	219.71	54.01
Acinetobacter baumannii-3GC	281.05	0.85	2.88	0.94	0.98	3.48	8.27	0.36	61.89	3.97	3.12	23.14	48.98	3.35	0.30	24.54	21.91	1.96	1.11	7.92	60.81	0.28
Citrobacter spp.-Aminoglycosides	32.19	0.51	0.03	0.13	0.35	0.27	0.38	0.31	0.84	0.41	1.10	0.14	1.40	0.62	0.06	20.70	1.87	0.17	0.05	0.50	0.79	1.57
Citrobacter spp.-BL-BLI	196.82	0.84	0.88	1.04	1.45	5.25	4.94	1.98	22.78	8.70	5.35	15.86	19.07	6.14	0.32	50.27	15.83	4.46	0.60	5.26	19.90	5.87
Citrobacter spp.-Carbapenems	188.77	0.50	0.17	0.62	3.12	2.02	3.95	0.59	25.33	2.39	2.05	2.16	3.19	5.32	0.15	106.79	14.48	2.18	0.83	3.68	5.45	3.78
Citrobacter spp.-Fluoroquinolones	207.74	1.19	0.18	0.82	2.85	2.41	4.74	1.25	16.91	3.37	6.38	3.32	10.06	9.59	0.27	109.83	16.75	3.69	0.52	4.32	5.11	4.13
Citrobacter spp.-4GC	93.90	0.42	0.11	0.57	0.38	0.43	2.20	1.34	4.59	0.65	3.99	0.18	0.82	2.94	0.22	44.24	17.74	1.30	0.32	2.88	1.47	7.11
Citrobacter spp.-Resistance to 1+	885.73	5.41	2.18	3.94	11.36	13.23	23.68	6.11	106.41	19.64	22.36	23.94	44.46	32.18	1.28	361.30	86.89	15.37	2.75	30.97	46.88	25.33
Citrobacter spp.-3GC	166.31	1.94	0.81	0.77	3.21	2.86	7.48	0.64	35.96	4.11	3.48	2.28	9.91	7.57	0.27	29.47	20.22	3.57	0.44	14.32	14.14	2.86
Enterobacter spp.-Aminoglycosides	106.23	0.50	0.10	0.40	1.41	3.08	3.58	1.18	5.56	2.72	4.13	0.71	3.85	3.66	0.20	49.28	9.51	0.83	0.25	7.07	2.51	5.67
Enterobacter spp.-Anti-pseudomonal	443.39	2.67	1.46	1.35	9.55	18.29	20.69	1.70	48.04	45.16	6.01	18.90	36.58	22.30	0.56	94.27	30.56	8.92	0.53	21.21	48.55	6.05
Enterobacter spp.-Carbapenems	616.42	3.08	1.10	2.30	4.44	7.94	21.37	2.08	66.25	17.31	4.64	16.65	24.19	21.08	0.69	300.94	47.12	12.68	1.92	21.90	31.98	6.66
Enterobacter spp.-Fluoroquinolones	267.53	2.05	0.29	2.43	2.73	7.80	7.54	2.60	28.68	4.85	9.42	3.69	9.61	10.19	0.55	102.99	36.04	4.12	0.76	7.55	11.94	11.76
Enterobacter spp.-4GC	185.74	2.13	0.10	1.55	3.52	4.52	7.11	3.02	5.56	13.93	11.80	1.08	1.91	5.29	0.50	61.69	18.78	4.02	0.41	16.96	11.24	10.64
Enterobacter spp.-Resistance to 1+	1790.81	11.71	3.71	9.40	25.48	47.43	66.26	12.42	172.98	94.55	41.90	43.19	82.82	70.32	2.81	632.90	168.95	33.73	6.59	99.27	116.27	48.07
Enterobacter spp.-TMP-SMX	171.51	1.27	0.66	1.36	3.84	5.80	5.97	1.84	18.88	10.58	5.91	2.16	6.68	7.81	0.31	23.73	26.93	3.16	2.73	24.58	10.05	7.29
Enterococcus faecalis-Fluoroquinolones	2125.48	11.73	5.48	9.70	27.65	59.27	61.69	10.46	151.91	147.21	37.57	49.72	155.97	26.06	1.57	778.25	176.59	30.74	5.74	103.71	231.92	42.28
Enterococcus faecalis-Resistance to 1+	2450.12	13.36	7.03	11.83	31.34	63.21	71.97	11.38	171.64	159.37	40.09	53.67	197.23	33.61	1.90	871.18	194.49	36.23	6.39	171.33	257.51	45.12
Enterococcus faecalis-Vancomycin	324.65	1.63	1.54	2.13	3.68	3.94	10.28	0.93	19.73	12.16	2.52	3.95	41.25	7.54	0.32	92.93	17.90	5.49	0.65	67.61	25.60	2.84
Enterococcus faecium-Fluoroquinol	3004.25	21.24	12.16	15.73	48.62	83.89	106.28	11.08	629.67	192.44	32.19	110.03	159.90	144.09	4.24	639.98	327.33	38.53	16.67	85.08	286.39	38.72

ones																						
Enterococcus faecium-Resistance to 1+	4199.71	31.93	17.70	22.72	61.98	125.01	144.12	14.49	687.74	229.57	42.72	126.85	397.95	246.44	6.18	879.81	376.53	72.77	18.50	293.36	352.99	50.31
Enterococcus faecium-Vancomycin	1195.46	10.69	5.55	6.99	13.35	41.12	37.83	3.41	58.07	37.12	10.53	16.82	238.05	102.35	1.94	239.83	49.20	34.24	1.83	208.28	66.60	11.60
Escherichia coli-Aminoglycosides	1307.00	24.09	3.73	6.04	23.55	25.84	30.72	9.02	62.60	84.85	23.06	57.17	106.89	39.58	1.06	476.81	104.12	14.37	1.61	41.48	125.00	45.26
Escherichia coli-Aminopenicillin	1417.18	4.65	8.73	8.38	18.45	43.43	47.75	4.92	196.66	48.93	24.66	52.90	185.48	35.26	2.14	83.02	79.71	47.75	11.45	162.34	335.20	15.45
Escherichia coli-BL-BLI	2942.22	31.82	30.65	23.64	45.43	85.60	102.80	13.68	235.05	127.47	44.35	132.11	450.47	65.27	3.01	247.39	204.52	113.07	11.39	340.35	591.90	42.53
Escherichia coli-Carbapenems	3616.50	9.19	5.97	9.58	35.27	15.91	39.35	10.79	110.99	109.26	35.76	24.02	40.00	125.80	2.26	2622.91	211.80	22.65	3.66	62.56	78.94	38.86
Escherichia coli-Fluoroquinolones	6871.04	57.98	13.34	36.95	83.85	123.17	329.48	25.37	783.84	315.50	76.20	315.55	493.75	195.63	4.69	2296.34	626.53	101.83	14.65	300.83	571.83	103.15
Escherichia coli-Resistance to 1+	26516.63	222.42	95.94	154.66	368.13	472.90	1154.19	115.58	2784.43	1400.19	398.67	931.58	1853.82	837.05	22.41	8269.02	2312.98	512.73	86.44	1489.47	2563.07	469.65
Escherichia coli-3GC	6909.24	54.52	19.59	45.28	102.18	105.69	432.83	26.93	1054.83	515.18	107.90	269.05	332.27	253.60	5.63	1861.35	782.45	145.58	19.70	228.77	416.58	129.21
Escherichia coli-TMP-SMX	3453.45	40.18	13.91	24.78	59.39	73.26	171.24	24.87	340.45	199.00	86.74	80.78	244.96	121.92	3.63	681.21	303.87	67.48	23.99	353.14	443.63	95.19
Group B Streptococcus-Fluoroquinolones	239.93	0.88	0.23	1.12	4.12	1.41	2.95	3.15	9.21	3.20	7.79	15.62	3.77	3.44	0.24	144.27	11.18	5.21	0.34	4.03	6.00	11.71
Group B Streptococcus-Macrolide	374.77	1.84	1.26	1.50	5.64	7.01	12.96	2.14	75.39	19.70	8.24	8.21	41.88	14.70	0.37	105.16	22.53	3.42	1.62	15.16	19.99	5.98
Group B Streptococcus-Penicillin	21.68	0.17	0.06	0.16	0.90	0.28	0.69	0.29	1.45	1.19	0.48	0.76	1.12	0.38	0.04	4.99	5.79	0.26	0.09	0.86	1.30	0.40
Group B Streptococcus-Resistance to 1+	636.38	2.90	1.55	2.79	10.66	8.70	16.61	5.59	86.05	24.09	16.51	24.59	46.78	18.52	0.64	254.41	39.49	8.89	2.06	20.06	27.29	18.10
Klebsiella pneumoniae-Aminoglycosides	976.26	15.34	1.39	4.57	13.94	35.74	30.03	6.37	41.99	97.21	22.92	10.44	35.96	36.81	0.83	384.33	42.46	20.80	1.52	74.87	74.52	24.09
Klebsiella pneumoniae-BL-BLI	408.70	4.78	2.84	2.62	5.51	13.62	23.99	1.87	20.24	16.62	5.34	26.01	49.62	13.65	0.33	45.97	18.78	9.71	1.00	43.22	97.17	5.82
Klebsiella pneumoniae-Carbapenems	2734.26	9.23	2.87	4.13	14.00	44.85	49.88	1.11	235.16	262.57	10.48	8.86	48.26	120.02	0.87	1369.65	216.20	36.25	5.35	190.11	90.99	12.67
Klebsiella pneumoniae-Fluoroquinolones	1236.85	11.23	1.45	6.98	18.14	43.06	37.16	5.27	137.93	107.14	16.79	13.49	43.46	54.88	1.31	361.57	116.43	31.86	3.84	95.72	107.12	21.98
Klebsiella pneumoniae-Resistance to 1+	8333.68	76.85	16.85	49.23	126.81	241.77	309.27	33.00	1010.54	656.00	118.01	129.78	311.93	378.87	7.60	2568.43	738.55	184.83	37.21	555.63	652.25	129.97

Klebsiella pneumoniae-3GC	1989.14	24.85	3.73	24.09	55.24	76.00	125.90	13.24	394.72	98.16	44.93	51.39	88.47	109.13	3.41	204.98	260.63	64.76	18.44	94.21	188.10	45.36
Klebsiella pneumoniae-TMP-SMX	988.48	11.42	4.57	6.83	19.99	28.51	42.31	5.13	180.51	74.30	17.56	19.59	46.16	44.39	0.85	201.92	84.05	21.45	7.06	57.50	94.34	20.04
Morganella spp.-Fluoroquinolones	427.09	3.83	0.14	2.18	3.85	3.80	21.93	2.87	35.32	3.76	8.97	1.50	11.05	17.76	0.55	248.81	17.44	2.70	2.22	16.53	12.24	9.58
Morganella spp.-4GC	153.92	0.46	0.13	1.00	1.08	1.14	1.27	2.49	9.39	1.92	7.19	0.84	1.97	4.70	0.54	89.24	8.25	0.95	0.41	10.19	3.44	7.30
Morganella spp.-Resistance to 1+	748.72	4.80	1.05	3.57	7.22	9.78	25.34	5.58	60.52	22.11	16.90	4.04	29.52	26.77	1.20	392.44	39.93	10.57	2.78	35.22	31.50	17.75
Morganella spp.-3GC	167.72	0.50	0.79	0.39	2.28	4.84	2.14	0.22	15.81	16.43	0.74	1.70	16.50	4.31	0.11	54.39	14.24	6.93	0.15	8.51	15.82	0.87
Other enterococci-Fluoroquinolones	3294.03	41.51	2.43	23.18	63.15	58.98	188.18	20.91	180.35	156.34	63.61	15.71	77.58	127.71	4.41	1300.74	378.51	80.74	19.55	310.78	97.00	82.68
Other enterococci-Resistance to 1+	3913.70	49.73	4.66	26.47	67.54	66.80	230.69	22.36	252.36	182.34	67.54	31.74	179.30	176.62	5.06	1370.41	441.36	117.67	21.42	382.60	130.38	86.66
Other enterococci-Vancomycin	619.67	8.23	2.22	3.29	4.39	7.82	42.51	1.45	72.01	26.01	3.92	16.03	101.73	48.91	0.64	69.68	62.85	36.93	1.86	71.82	33.39	3.97
Proteus spp.-Aminoglycosides	175.82	0.71	0.18	0.48	2.54	1.69	1.76	1.22	7.45	20.53	5.77	1.17	10.25	9.09	0.12	87.06	8.57	1.57	0.20	3.68	7.44	4.29
Proteus spp.-Aminopenicillin	293.43	2.89	1.12	1.90	3.84	7.11	10.73	1.74	23.17	18.80	5.35	6.35	11.84	7.69	0.31	73.27	25.96	8.86	1.63	31.60	43.66	5.63
Proteus spp.-Fluoroquinolones	621.50	4.05	0.33	2.14	5.34	9.67	11.22	2.97	74.04	12.72	12.22	13.05	62.34	19.74	0.39	277.10	25.68	12.19	0.59	41.60	23.10	10.87
Proteus spp.-Resistance to 1+	2362.36	18.47	4.72	11.73	33.81	52.83	64.07	13.98	284.30	78.20	47.24	78.22	155.14	105.52	2.56	781.23	178.89	52.01	9.32	164.18	175.53	50.22
Proteus spp.-3GC	943.46	8.31	2.65	4.77	17.18	28.27	27.07	5.08	156.25	13.88	12.93	47.39	47.84	53.92	1.40	260.18	84.35	24.03	4.18	66.03	58.52	19.19
Proteus spp.-TMP-SMX	328.15	2.52	0.45	2.44	4.91	6.09	13.28	2.96	23.39	12.28	10.97	10.25	22.87	15.07	0.34	83.61	34.33	5.37	2.71	21.27	42.82	10.24
Pseudomonas aeruginosa-Aminoglycosides	144.97	1.44	0.10	0.78	2.14	5.80	6.51	0.49	6.21	17.09	1.48	2.27	6.21	8.54	0.14	53.74	12.26	2.94	0.20	5.91	7.06	3.65
Pseudomonas aeruginosa-Anti-pseudomonal	497.06	3.79	2.26	4.00	12.63	21.04	20.80	4.36	62.53	37.98	9.62	35.55	34.67	31.04	0.88	52.54	47.31	12.28	2.18	25.98	63.78	11.92
Pseudomonas aeruginosa-Carbapenems	2116.00	19.34	4.01	11.77	26.31	65.06	109.64	3.98	265.87	173.74	8.49	32.48	110.07	92.49	2.08	720.68	146.54	41.93	9.36	115.62	139.48	16.86
Pseudomonas aeruginosa-Fluoroquinolones	979.04	9.52	1.54	4.26	17.03	37.80	44.56	2.69	98.27	98.86	8.29	19.87	88.08	52.50	0.84	259.34	50.95	22.96	3.36	71.01	72.74	14.44
Pseudomonas aeruginosa-4GC	211.89	1.06	0.08	0.46	3.35	5.69	5.23	0.69	13.14	9.52	2.32	4.50	14.47	11.23	0.18	105.42	12.69	3.55	0.53	6.10	8.55	3.08
Pseudomonas aeruginosa-Resistance to 1+	4423.08	37.39	8.73	23.05	70.28	142.79	196.24	14.42	596.13	356.54	43.96	101.01	298.90	243.64	4.39	1286.04	297.15	88.51	16.23	237.11	302.88	57.31

Pseudomonas aeruginosa-3GC	474.11	2.24	0.74	1.77	8.81	7.40	9.51	2.21	150.10	19.35	13.77	6.36	45.40	47.85	0.27	94.32	27.39	4.84	0.59	12.49	11.27	7.36
Serratia spp.-Aminoglycosides	30.91	0.26	0.01	0.15	0.63	0.54	0.91	0.56	1.53	4.12	1.88	0.14	0.71	1.05	0.08	10.64	2.22	0.69	0.46	1.47	0.34	2.52
Serratia spp.-Anti-pseudomonal	98.95	0.75	0.06	0.38	2.09	1.30	2.64	0.84	12.09	8.60	4.63	3.03	4.24	3.86	0.14	33.51	7.00	3.16	0.21	5.33	2.49	2.59
Serratia spp.-Carbapenems	112.54	0.63	0.27	0.77	1.54	1.43	4.11	0.62	7.71	8.08	1.06	1.95	2.62	4.17	0.54	47.89	12.54	1.08	0.34	11.48	2.26	1.46
Serratia spp.-Fluoroquinolones	38.45	0.24	0.09	0.22	0.66	0.47	1.12	0.47	3.45	0.59	1.80	0.72	2.24	1.51	0.10	13.22	5.20	0.87	0.23	1.98	1.12	2.15
Serratia spp.-4GC	70.74	0.44	0.02	0.45	2.01	4.51	1.35	2.59	3.21	7.12	5.01	0.10	0.23	3.36	0.19	17.32	4.12	1.72	0.24	4.11	0.72	11.93
Serratia spp.-Resistance to 1+	397.74	3.27	0.56	2.47	8.14	8.96	13.53	5.37	33.61	31.17	15.86	8.05	11.76	16.61	1.19	126.34	35.55	9.03	1.58	29.77	12.23	22.71
Serratia spp.-3GC	46.15	0.96	0.10	0.51	1.21	0.72	3.40	0.30	5.62	2.65	1.49	2.11	1.73	2.67	0.13	3.75	4.47	1.50	0.10	5.40	5.30	2.05
Staphylococcus aureus-Fluoroquinolones	354.11	3.03	0.45	1.37	4.28	4.20	10.39	1.42	29.33	12.91	3.91	12.68	26.61	8.04	0.37	171.46	26.58	3.51	0.84	10.12	20.13	2.41
Staphylococcus aureus-Macrolide	371.77	3.43	0.70	2.10	5.71	8.22	13.99	1.24	71.51	7.38	5.30	11.83	31.07	17.11	0.45	100.63	17.83	6.03	1.83	34.42	24.60	6.36
Staphylococcus aureus-Methicillin	2415.20	24.93	4.48	17.25	28.25	39.08	122.32	8.50	309.45	78.54	25.77	89.16	180.62	134.81	3.24	719.92	259.87	61.82	9.03	143.94	113.40	40.87
Staphylococcus aureus-Resistance to 1+	3490.46	32.31	6.22	22.88	42.18	53.75	151.88	17.45	429.11	104.24	58.04	116.06	245.66	173.08	4.44	1165.19	343.51	73.20	14.92	194.58	167.13	74.61
Staphylococcus aureus-TMP-SMX	288.10	0.47	0.37	1.77	3.10	1.25	2.58	6.00	13.02	2.25	22.07	1.00	4.57	10.85	0.31	157.61	26.44	0.84	2.96	2.87	4.45	23.29
Staphylococcus aureus-Vancomycin	61.27	0.45	0.21	0.38	0.82	0.99	2.60	0.29	5.79	3.17	0.98	1.38	2.79	2.27	0.08	15.57	12.78	1.00	0.27	3.23	4.56	1.68

GBD= Global Burden of Diseases, Injuries, and Risk Factors Study. 3GC=third-generation cephalosporins. 4GC=fourth-generation cephalosporins. Anti-pseudomonal=anti-pseudomonal penicillin or beta-lactamase inhibitors. BL-BLI=β-lactam or β-lactamase inhibitors. Resistance to 1+=resistance to one or more drugs. TMP-SMX= trimethoprim/sulfamethoxazole. AMR=antimicrobial resistance. GBD= Global Burden of Diseases, Injuries, and Risk Factors Study.

Figure S1. All-age rate of DALYs attributable to and associated with bacterial AMR among 21 GBD regions, 2019.

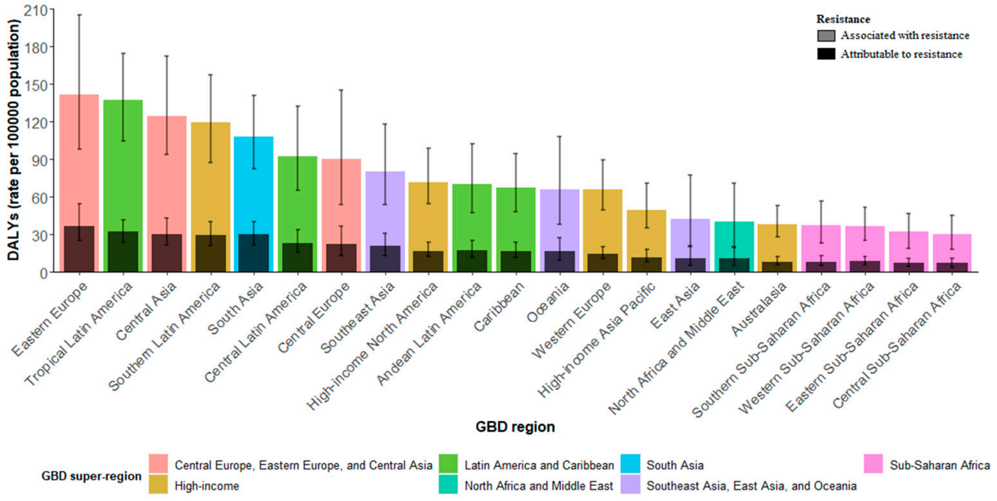

DALYs=disability-adjusted life-years. AMR=antimicrobial resistance. GBD= Global Burden of Diseases, Injuries, and Risk Factors Study. Error bars indicate 95% uncertainty intervals.

Figure S2. All-age count of DALYs attributable to and associated with bacterial AMR globally among 14 pathogens, 2019.

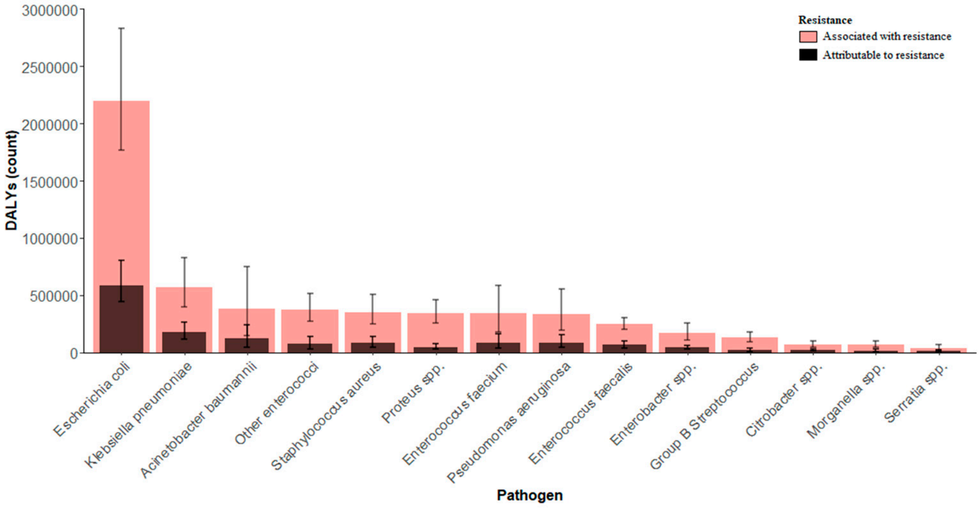

DALYs=disability-adjusted life-years. AMR=antimicrobial resistance. Error bars indicate 95% uncertainty intervals.

Figure S3. Fraction of DALYs attributable to (A) and associated with (B) bacterial AMR among 21 GBD regions and 14 pathogens, 2019.

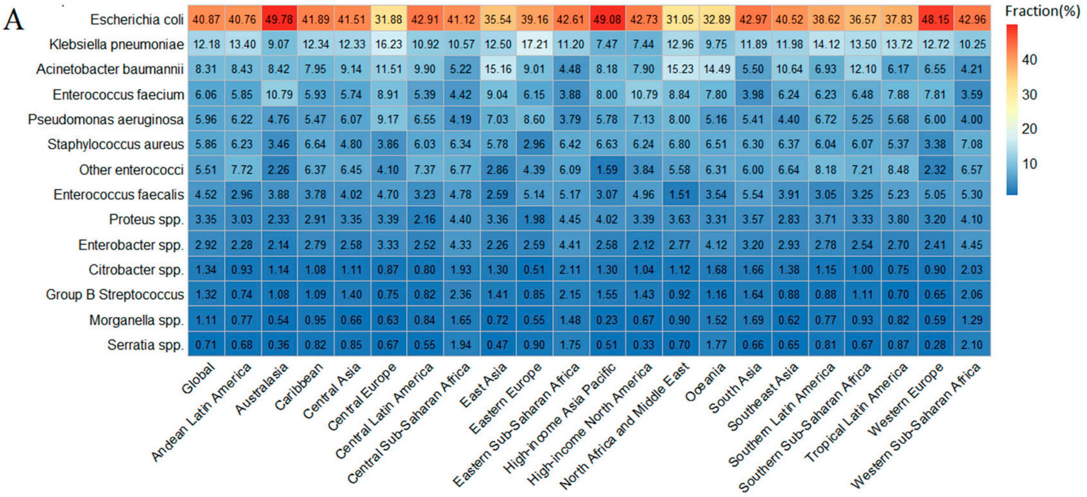

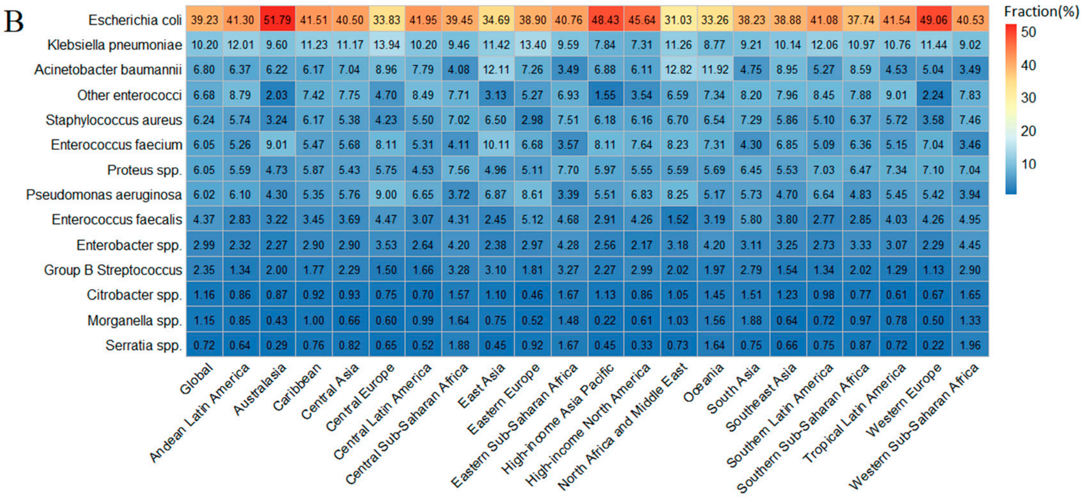

DALYs=disability-adjusted life-years. AMR=antimicrobial resistance. GBD= Global Burden of Diseases, Injuries, and Risk Factors Study.

Figure S4. All-age count of DALYs attributable to and associated with bacterial AMR globally among 13 antibiotic classes, 2019.

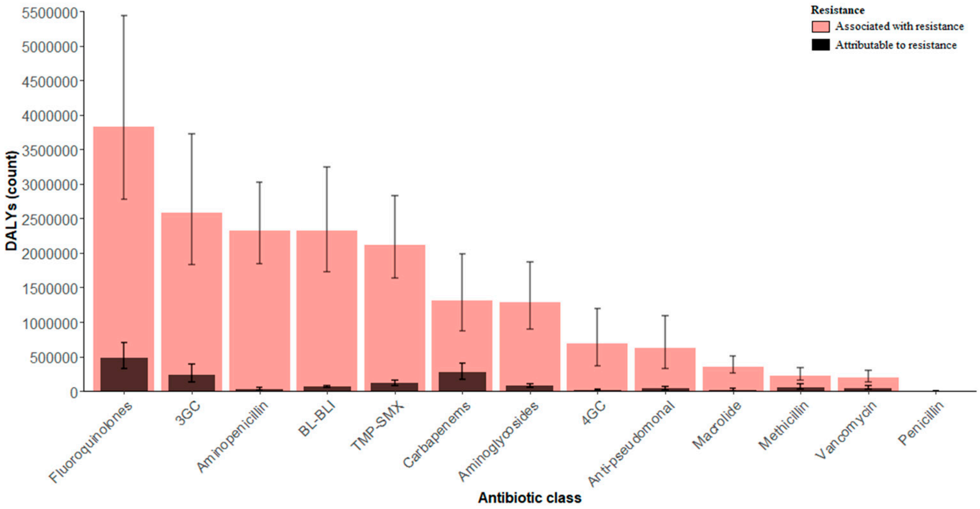

DALYs=disability-adjusted life-years. 3GC=third-generation cephalosporins. 4GC=fourth-generation cephalosporins. Anti-pseudomonal=anti-pseudomonal penicillin or beta-lactamase inhibitors. BL-BLI=β-lactam or β-lactamase inhibitors. Resistance to 1+=resistance to one or more drugs. TMP-SMX=trimethoprim/sulfamethoxazole . AMR=antimicrobial resistance. Error bars indicate 95% uncertainty intervals.

Figure S5. Fraction of DALYs attributable to (A) and associated with (B) bacterial AMR among 21 GBD regions and 13 antibiotic classes, 2019.

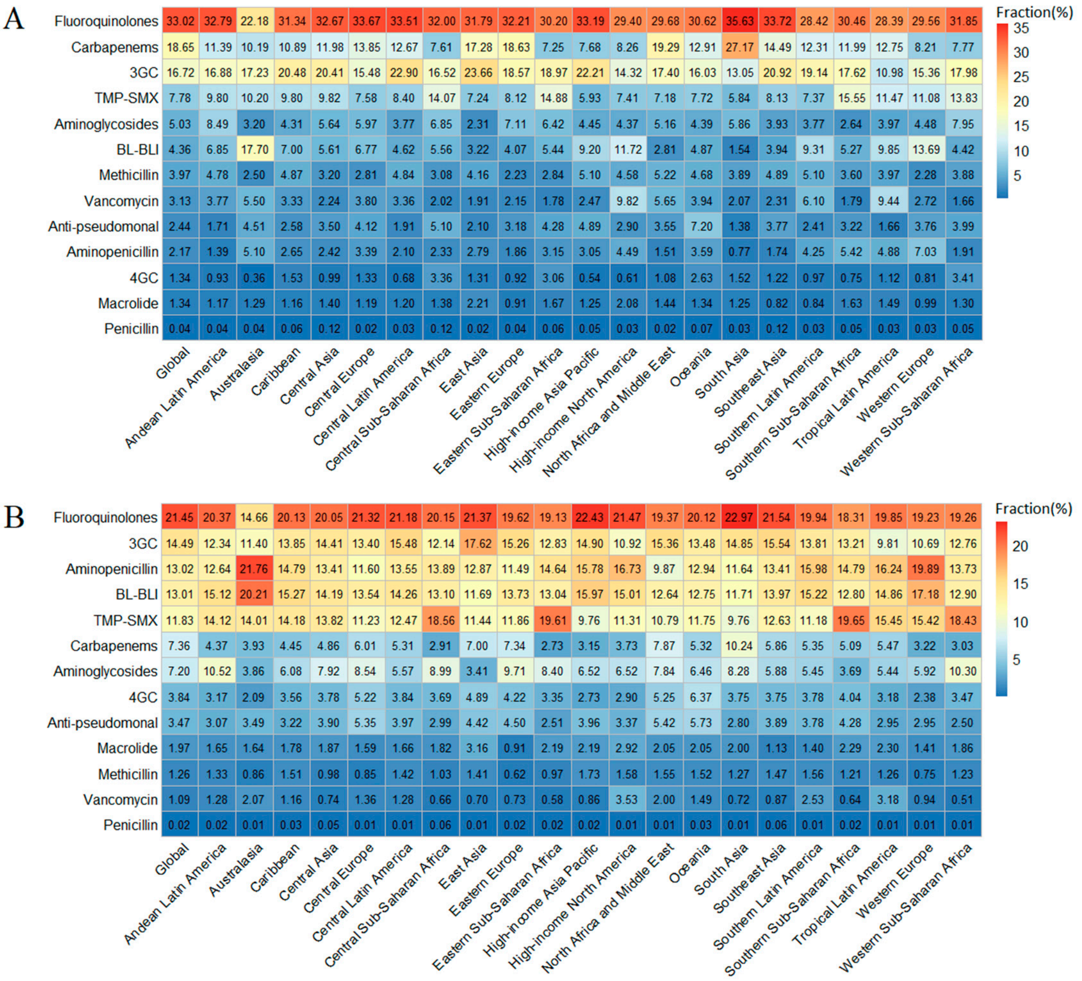

DALYs=disability-adjusted life-years. 3GC=third-generation cephalosporins. 4GC=fourth-generation cephalosporins. Anti-pseudomonal=anti-pseudomonal penicillin or beta-lactamase inhibitors. BL-BLI=β-lactam or β-lactamase inhibitors. Resistance to 1+=resistance to one or more drugs. TMP-SMX=trimethoprim/sulfamethoxazole . AMR=antimicrobial resistance. GBD= Global Burden of Diseases, Injuries, and Risk Factors Study.
